# Diverse Large HIV-1 Non-subtype B Clusters Are Spreading Among Men Who Have Sex With Men in Spain

**DOI:** 10.3389/fmicb.2019.00655

**Published:** 2019-04-03

**Authors:** Elena Delgado, Sonia Benito, Vanessa Montero, María Teresa Cuevas, Aurora Fernández-García, Mónica Sánchez-Martínez, Elena García-Bodas, Francisco Díez-Fuertes, Horacio Gil, Javier Cañada, Cristina Carrera, Jesús Martínez-López, Marcos Sintes, Lucía Pérez-Álvarez, Michael M. Thomson, The Spanish Group for the Study of New HIV Diagnoses, Josefa Muñoz

**Affiliations:** **Basque Country**: Hospital Universitario de Basurto, Bilbao; Hospital Universitario de Cruces, Bilbao; Hospital de Galdakao; Hospital Universitario Donostia, San Sebastián; Hospital Universitario de Álava, Vitoria: **Galicia**: Complejo Hospitalario universitario de Ferrol, Ferrol A Coruña; Complejo Hospitalario Universitario de A Coruña; Hospital Universitario Lucus Augusti, Lugo; Complejo Hospitalario Universitario de Ourense; Complejo Hospitalario Universitario de Vigo, Vigo Pontevedra; Complejo Hospitalario de Pontevedra: **Navarra**: Complejo Hospitalario de Navarra, Pamplona: **Madrid**: Centro Sanitario Sandoval, Madrid; Hospital de Fuenlabrada; Hospital Clínico Universitario San Carlos, Madrid; Fundación Jiménez Díaz, Madrid; Hospital Severo Ochoa, Leganés; Hospital Universitario Puerta de Hierro, Majadahonda: **Castilla y León**: Hospital Clínico Universitario de Valladolid; Hospital Río Hortega, Valladolid; Hospital Virgen de la Concha, Zamora: **La Rioja**: Hospital San Pedro: **Aragón**: Hospital Universitario Miguel Servet, Zaragoza: **Castilla-La Mancha**: Hospital Virgen de la Salud, Toledo: **Comunitat Valenciana**: Hospital Universitari Sant Joan d’Alacant; ^1^HIV Biology and Variability Unit, Centro Nacional de Microbiología, Instituto de Salud Carlos III, Madrid, Spain; ^2^CIBER de Epidemiología y Salud Pública (CIBERESP), Madrid, Spain; ^3^AIDS Immunopathogenesis Unit, Centro Nacional de Microbiología, Instituto de Salud Carlos III, Madrid, Spain; ^4^European Program for Public Health Microbiology Training, European Centre for Disease Prevention and Control, Stockholm, Sweden

**Keywords:** HIV-1, molecular epidemiology, phylogeny, phylodynamics, men who have sex with men, subtypes, circulating recombinant forms, clusters

## Abstract

In Western Europe, the HIV-1 epidemic among men who have sex with men (MSM) is dominated by subtype B. However, recently, other genetic forms have been reported to circulate in this population, as evidenced by their grouping in clusters predominantly comprising European individuals. Here we describe four large HIV-1 non-subtype B clusters spreading among MSM in Spain. Samples were collected in 9 regions. A pol fragment was amplified from plasma RNA or blood-extracted DNA. Phylogenetic analyses were performed via maximum likelihood, including database sequences of the same genetic forms as the identified clusters. Times and locations of the most recent common ancestors (MRCA) of clusters were estimated with a Bayesian method. Five large non-subtype B clusters associated with MSM were identified. The largest one, of F1 subtype, was reported previously. The other four were of CRF02_AG (CRF02_1; *n* = 115) and subtypes A1 (A1_1; *n* = 66), F1 (F1_3; *n* = 36), and C (C_7; *n* = 17). Most individuals belonging to them had been diagnosed of HIV-1 infection in the last 10 years. Each cluster comprised viruses from 3 to 8 Spanish regions and also comprised or was related to viruses from other countries: CRF02_1 comprised a Japanese subcluster and viruses from 8 other countries from Western Europe, Asia, and South America; A1_1 comprised viruses from Portugal, United Kingom, and United States, and was related to the A1 strain circulating in Greece, Albania and Cyprus; F1_3 was related to viruses from Romania; and C_7 comprised viruses from Portugal and was related to a virus from Mozambique. A subcluster within CRF02_1 was associated with heterosexual transmission. Near full-length genomes of each cluster were of uniform genetic form. Times of MRCAs of CRF02_1, A1_1, F1_3, and C_7 were estimated around 1986, 1989, 2013, and 1983, respectively. MRCA locations for CRF02_1 and A1_1 were uncertain (however initial expansions in Spain in Madrid and Vigo, respectively, were estimated) and were most probable in Bilbao, Spain, for F1_3 and Portugal for C_7. These results show that the HIV-1 epidemic among MSM in Spain is becoming increasingly diverse through the expansion of diverse non-subtype B clusters, comprising or related to viruses circulating in other countries.

## Introduction

HIV-1 exhibits a characteristic high genetic variability, derived from elevated mutation and recombination rates. Through these mechanisms, the main (M) HIV-1 group, causative of the pandemic, has evolved into multiple genetic forms, designated subtypes, of which nine have been identified, subsubtypes, circulating recombinant forms (CRFs), of which 93 are currently recognized (HIV Sequence Database, 2019; Reis et al., 2019), and unique recombinant forms (URFs). The most globally prevalent HIV-1 genetic form is subtype C, estimated to represent around 47% worldwide infections, followed, in this order, by subtype B, subtype A, CRF02_AG, CRF01_AE, subtype G, and subtype D, with each of the remaining genetic forms estimated to represent less than 1% of global infections (Hemelaar et al., 2018).

In Western Europe, the predominant HIV-1 genetic form is subtype B, which was initially introduced among MSM and persons who inject drugs (PWID) (Lukashov et al., 1996; Casado et al., 2000; Kuiken et al., 2000; Hué et al., 2005; Beloukas et al., 2016). In early descriptions of HIV-1 genetic diversity in Western Europe, non-subtype B genetic forms were restricted to heterosexually infected immigrants coming from areas where those clades predominate, mainly sub-Saharan Africans, and European individuals epidemiologically linked to people from such areas (Fransen et al., 1996; Op de Coul et al., 1998; Thomson and Nájera, 2001). The first reports of HIV-1 non-subtype B genetic forms circulating in Western Europe among individuals without known epidemiological links to other geographic areas described the circulation of CRF01_AE among PWID in Finland (Liitsola et al., 2000) and of subtype G and CRF14_BG among a minority of HIV-1-infected PWID in the region of Galicia, Northwest Spain (Thomson et al., 2001; Delgado et al., 2002). Subsequent studies showed that the genetic forms circulating in Galicia derived from a subtype G variant widely circulating in Portugal, transmitted both through sexual contact and among PWID (Esteves et al., 2002, 2003; Palma et al., 2007; Carvalho et al., 2015). In recent years, an increasing prevalence of non-subtype B infections has been observed in Western Europe, reflecting both their importation from other geographical areas and their circulation among the local population (Abecasis et al., 2013; Beloukas et al., 2016; Hemelaar et al., 2018; Paraskevis et al., 2019).

In the current HIV-1 epidemic in Western Europe, the predominant propagation mode is through sexual contact among MSM (European Centre for Disease Prevention and Control, 2017 and WHO Regional Office for Europe 2017; Núñez et al., 2018), a population in which a resurgence of the HIV-1 epidemic has been observed since the 2000s, which is part of a global phenomenon (Bezemer et al., 2008; Beyrer et al., 2012). This has been accompanied by the emergence of phylogenetically identifiable transmission clusters, whose expansion is mostly driven by individuals with recent infection who are unaware of their HIV status (Lewis et al., 2008; Cuevas et al., 2009; Bezemer et al., 2010; Chalmet et al., 2010; Fisher et al., 2010; Zehender et al., 2010; Ambrosioni et al., 2012; Frange et al., 2012; Thomson et al., 2012; Audelin et al., 2013; Delgado et al., 2015; Esbjörnsson et al., 2016; Hoenigl et al., 2016; Chaillon et al., 2017; Patiño-Galindo et al., 2017; Parczewski et al., 2017; Verhofstede et al., 2018; Paraskevis et al., 2019). As expected, most clusters are of subtype B, but multiple instances of propagation of other HIV-1 genetic forms among European MSM have also been reported. These include subtypes A1 (Lai et al., 2016; Ragonnet-Cronin et al., 2016), C (de Oliveira et al., 2010; Lai et al., 2014; Ragonnet-Cronin et al., 2016), and F1 (Castro et al., 2010; Lai et al., 2012; Thomson et al., 2012; Delgado et al., 2015; Vinken et al., 2017; Verhofstede et al., 2018); CRF01_AE (von Wyl et al., 2011), CRF02_AG (Giuliani et al., 2013; Brand et al., 2014; Dauwe et al., 2015; Tamalet et al., 2015; Chaillon et al., 2017; Verhofstede et al., 2018), CRF17_BF (Fabeni et al., 2015), CRF19_cpx (Patiño-Galindo et al., 2015; González-Domenech et al., 2018; Pérez-Parra et al., 2018), CRF50_A1D (Foster et al., 2014), CRF56_cpx (Leoz et al., 2013), and CRF60_BC (Monno et al., 2012; Simonetti et al., 2014). However, the expansion of these clades has had a limited impact on the overall genetic composition of the HIV-1 epidemic among MSM in Western Europe, which is still largely dominated by subtype B. The only exception, though geographically restricted, is a large F1 subtype cluster of Brazilian ancestry, which represented a substantial proportion of new HIV-1 diagnoses among MSM in Northwest Spain (Thomson et al., 2012; Delgado et al., 2015). Here we describe four additional large non-subtype B clusters expanding among MSM in Spain, of CRF02_AG and of subtypes A1, F1 and C, each circulating in several Spanish regions and related to viruses from other countries.

## Materials and Methods

### Samples

Plasma or whole blood samples were collected from 1999 to 2018 from HIV-1-infected individuals attended at hospitals from 15 provinces from 9 regions of Spain (Basque Country, Galicia, Navarre, Castilla y León, La Rioja, Madrid, Castilla-La Mancha, Aragón, and Comunidad Valenciana). The regional sample representativeness is variable, being the greatest in the regions of Basque Country, where all public hospitals participated, and Galicia, where all but one public hospitals participated. The study was approved by the Bioethics and Animal Well-being Committee of Instituto de Salud Carlos III, Majadahonda, Madrid, Spain. Written informed consent was obtained from all participants in the study.

### RNA and DNA Extraction, RT-PCR Amplification, and Sequencing

Amplification of HIV-1 fragments was done either from plasma RNA or from DNA extracted from whole blood. RNA was extracted from 1 ml plasma using Nuclisens EasyMAG kit (bioMérieux, Marcy l’Etoile, France), following the manufacturer’s instructions. DNA was extracted from 200 μl blood using QIAmp DNA Blood mini kit (Qiagen, Hilden, Germany), following the manufacturer’s instructions. An HIV-1 PR-RT fragment (approximately 1.4 kb) was amplified by RT-PCR followed by nested PCR, in the case of RNA, or by nested PCR, in the case of DNA, using previously reported primers (Delgado et al., 2015). In selected samples, NFLG amplification was done in four overlapping segments, as described (Delgado et al., 2002; Sierra et al., 2005), using RNA extracted either from plasma or from the primary isolate’s culture supernatant grown from plasma using a previously described protocol (Delgado et al., 2012). Direct sequencing of the amplified products was done using an automated capillary sequencer. Sequence electropherograms were assembled and edited with Seqman (DNASTAR, Madison, WI, United States). Newly obtained sequences are deposited in GenBank under accessions MK177651-MK177824 (PR-RT sequences) and MK177825-MK177829, KT276258, KY496622, and KY989952 (NFLG sequences).

### Phylogenetic Sequence Analyses

Sequences were aligned with MAFFT v.7 (Katoh and Standley, 2013). Initial trees with all sequences were constructed with the approximate ML method implemented in FastTree v2.1.10 (Price et al., 2010) using the general time reversible (GTR)+CAT evolutionary model and assessment of node support with Shimodaira-Hasegawa (SH)-like local support values. Transmission clusters were defined as those supported by SH-like values ≥ 0.95 comprising four or more individuals, with a majority of them being native Spanish. To determine whether the identified clusters were still supported when globally sampled sequences were included and to identify viruses from other areas belonging or phylogenetically related to them, the analyses were repeated including all PR-RT sequences of 1 kb or longer of the same genetic form available at the Los Alamos HIV Sequence Database (HIV Sequence Database, 2019), downloaded with the option “one sequence per patient.” Support for clusters thus identified with FastTree was subsequently assessed with phylogenetic trees constructed via ML with PhyML v3.0 (Guindon et al., 2005), using the best-fit evolutionary model selected by Smart Model Selection (SMS) program (Lefort et al., 2017) and heuristic searches based on subtree pruning and regrafting (SPR) moves, with estimation of node support by the approximate likelihood ratio test (aLRT), SH-like procedure (Anisimova and Gascuel, 2006; Guindon et al., 2010). To keep computational times reasonable, in the analyses with PhyML only several hundred sequences (around 200–400) branching most closely to the clusters of interest in the previous FastTree analyses, together with PR-RT sequences from NFLG from databases, were included. Clusters were confirmed if in the analyses including database sequences with both FastTree and PhyML their node support values were ≥ 0.95. Trees were visualized with Dendroscope 3 (Huson and Scornavacca, 2012) or FigTree v1.4.2^1^. Intersubtype recombination was analyzed by bootscanning with Simplot v3.5 (Lole et al., 1999), with tree construction with the neighbor-joining method, using the Kimura 2-parameter substitution model and windows of 400 or 600 nt moving in 20 nt steps.

### Antiretroviral Drug Resistance Determination

Antiretroviral (ARV) drug resistance was analyzed with the Calibrated Population Resistance Tool (Gifford et al., 2009).

### Temporal and Geographic Estimations of Cluster Origins

To estimate times of the most recent common ancestors (tMRCA) of clusters and their most probable geographical locations, a Bayesian MCMC coalescent method as implemented in BEAST v1.8.1 (Drummond et al., 2012) was used. Prior to these analyses, the existence of temporal signal in the datasets was assessed by an analysis of root-to-tip distances against dates of sampling using TempEst (Rambaut et al., 2016). For the BEAST analyses, we used all PR-RT sequences ≥ 1 kb from each cluster and related sequences, as determined in the ML phylogenetic analyses, excluding sequences without data on year or location of sample collection. PR-RT sequences derived from NFLG sequences of the corresponding genetic form downloaded from the HIV Sequence Database were also included in these analyses, using not more than 5 sequences per country of sampling. In the case of the CRF02_1 cluster, 40 randomly selected CRF02_AG PR-RT sequences lacking drug resistance mutations downloaded from the Los Alamos HIV Sequences database were included, since using those derived from NFLG resulted in relatively low *r*^2^ values in the TempEst analysis. We chose an HKY substitution model with gamma-distributed among-site rate heterogeneity and two partitions in codon positions (1st+2nd; 3rd) (Shapiro et al., 2006); uniform priors were used for absolute substitution rates (0-0.02 sub/site/year) and relative substitution rates in codon positions 1st+2nd and 3rd (0-10); we also used an uncorrelated lognormal relaxed clock model and a Bayesian skyline plot demographic model (Drummond et al., 2005). Each MCMC chain was run for 100 million to 200 million generations, sampling every 5,000 generations. MCMC convergence and effective sample sizes (ESS) were checked with Tracer v.1.6^2^, ensuring that the ESS of each parameter was > 200. Results were summarized with a maximum clade credibility (MCC) tree, using TreeAnnotator v1.8.1, after removal of a 50% burn-in. The MCC trees were visualized with FigTree v1.4.2.^1^ Parameter uncertainty was summarized in the 95% highest posterior density (HPD) intervals.

### Statistical Analyses

Differences in clustering frequency between MSM and heterosexually infected individuals and changes in proportions of non-subtype B infections along time in newly diagnosed sexually infected individuals were analyzed with Fisher’s exact test. Only native Spish individuals were included in these analyses in order to focus on locally circulating strains, minimizing the confounding effect of imported HIV-1 variants acquired in other countries. Numbers of individuals used in these analyses were 7080 in the first and 2060 in the second.

## Results

Using HIV-1 PR-RT sequences from 10,506 individuals obtained by us (whose data are summarized in Supplementary Table 1), we identified 320 phylogenetic clusters comprising 4 or more individuals, 247 of subtype B and 73 of other genetic forms. Belonging to a cluster was more frequent among Spanish MSM than among heterosexually-infected Spanish individuals (68% vs. 31%; *p* = 7.7 × 10^-6^) (Supplementary Figure 1). Differences were also significant when subtype B and non-subtype B clusters were analyzed separately. An increase along time of non-subtype B infections among newly diagnosed Spanish individuals was also observed. Statistically significant increases were observed in Spanish MSM from 2005–2009 to 2010–2014 (from 10.9% to 26.7%; *p* = 1.4 × 10^-6^) and among heterosexually infected Spanish individuals from 2010–2014 to 2015–2018 (from 28.5% to 39.7%; *p* = 0.0097) (Supplementary Figure 2).

Of the non-subtype B clusters, 5 large ones (here defined as those comprising 10 or more individuals) were associated with MSM. The largest one, of F1 subtype (currently comprising 187 individuals), was reported previously (Thomson et al., 2012; Delgado et al., 2015). The other four were of CRF02_AG and of subtypes A1, F1, and C, henceforth designated CRF02_1, A1_1, F1_3, and C_7, respectively. All four clusters were well supported when the analyses were repeated with FastTree including all PR-RT sequences > 1 kb of the respective genetic forms available at the Los Alamos HIV Sequence database (with numbers from 1,318 of subtype F1 to 22,762 of subtype C) and with PhyML including several hundred (from 312 for subtype C to 513 for CRF02_AG) database sequences branching closer to each cluster in the FastTree analysis and derived from NFLG sequences. These analyses also allowed to identify viruses from databases belonging or closely related to the clusters. Epidemiological data of samples studied by us belonging to these clusters and to subclusters within them are shown in Table 1.

**Table 1:** Epidemiological data of clusters^∗^.

Cluster/ Subcluster	N	Gender			Transmission route					Region									Country of origin^†^				Years of HIV-1 diagnosis
		M	F	ND	MSM	MSx	HT	PWID	ND	BC	GA	NA	MD	AR	LR	CV	CL	CM	Spain	LAm	Other	ND
CRF02_1	67	58	8	1	30	12	18	1	6	31	3	3	10	15		3	1	1	53	10	4		2008–2018
1	49	47	1	1	28	12	5	1	3	26	3	1	9	6		3		1	40	7	2		2008–2018
1_1	17	17			11	4	1		1	15	2								14	2	1		2008–2018
1_2	7	7			3	1	3						1	6					6	1			2016–2018
3	10	5	5				10							9			1		8	1	1		2016–2018
A1_1	54	50	3	1	36	6	6		6	6	22	18	3		5				37	10	5	2	2006–2018
1	30	27	3		18	4	3		5	6		18	1		5				19	5	5	1	2013–2017
2	16	15		1	12	1	2	1			15		1						13	2		1	2009–2018
2_1	10	10			8		1		1		9		1										2016–2018
F1_3	36	36			17	10	3		6	30		3	3						29	6	1		2014–2018
C_7	14	14			5	6	3			4	9						1		10	1		3	2009–2017
1	8	8			5	2	1				7								6	1		1	2012–2017
2	4	4				3	1			4									3			1	2009–2015

*^∗^Only data from samples processed by the authors are included. ^†^Country of which the individual is native. ND, no data. HT, heterosexual. MSx, male, unspecified sexual. BC, Basque Country. MD, Madrid. GA, Galicia. NA, Navarre. AR, Aragon. CL, Castilla y León. CM, Castilla-La Mancha. CV, Comunidad Valenciana. LR, La Rioja. LAm, Latin American country.*

### CRF02_1 Cluster

**FIGURE 1:**
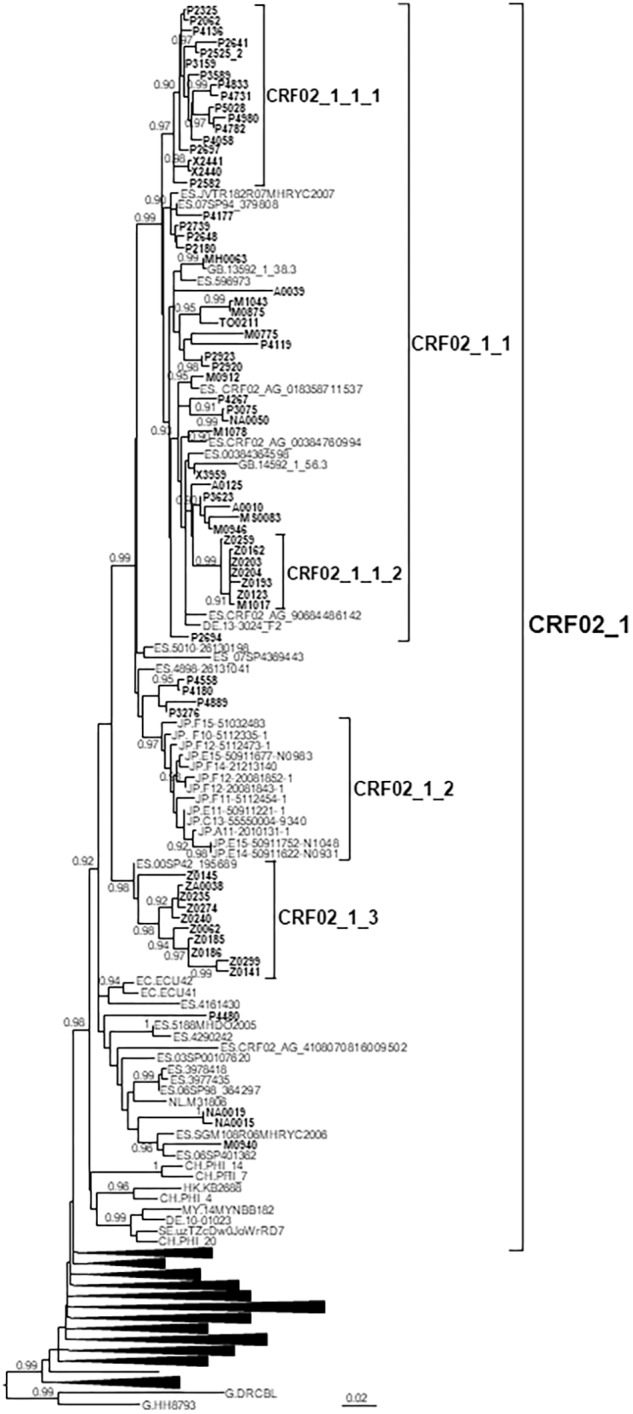
Maximum likelihood tree of PR-RT sequences of the CRF02_1 cluster. The tree was constructed with PhyML, with assessment of node support with the aLRT SH-like procedure. The analysis incorporates 513 CRF02_AG PR-RT sequences from databases that in preliminary analyses with FastTree branched closer to the CRF02_1 cluster and from NFLG sequences, and two subtype G sequences used to root the tree. For better viewing, clades outside of the CRF02_1 cluster are collapsed. Only aLRT SH-like node support values ≥ 0.9 are shown. Sequences obtained by us are in bold type. Sequences from databases are labeled with the two-letter ISO code of the country of sample collection followed by the sample name.

CRF02_1 comprised 115 individuals, 67 studied by us and 48 whose sequences were retrieved from databases (Figure 1). Most samples were collected in Spain, but there were also samples from Japan, Switzerland, United Kingdom, Ecuador, Netherlands, Sweden, Germany, Malaysia, and Hong Kong. Samples from Spain were from 8 regions, mainly from Basque Country, Madrid, and Aragon.

Years of HIV-1 diagnosis were 2008–2018.

CRF02_1 comprised three subclusters, designated with numerals 1-3. CRF02_1_1 comprised 60 individuals from 7 Spanish regions, two from United Kingdom and one from Germany. Within it, two subsubclusters were distinguished, associated with the Basque Country and the city of Zaragoza, Aragón, respectively; CRF02_1_2 comprised 13 individuals from Japan; and CRF02_1_3 comprised 11 individuals, 10 of them from Zaragoza.

Among samples studied by us, 88% were from men. Transmission route was sexual in the great majority, and, among those infected via sexual contact, 51% were self-declared MSM. Interestingly, all 10 individuals from subcluster CRF02_1_3 studied by us (5 women and 5 men, all from Zaragoza) were infected via heterosexual contact.

Most individuals in this and in the other clusters here described were native Spanish (Table 1).

### A1_1 Cluster

**FIGURE 2:**
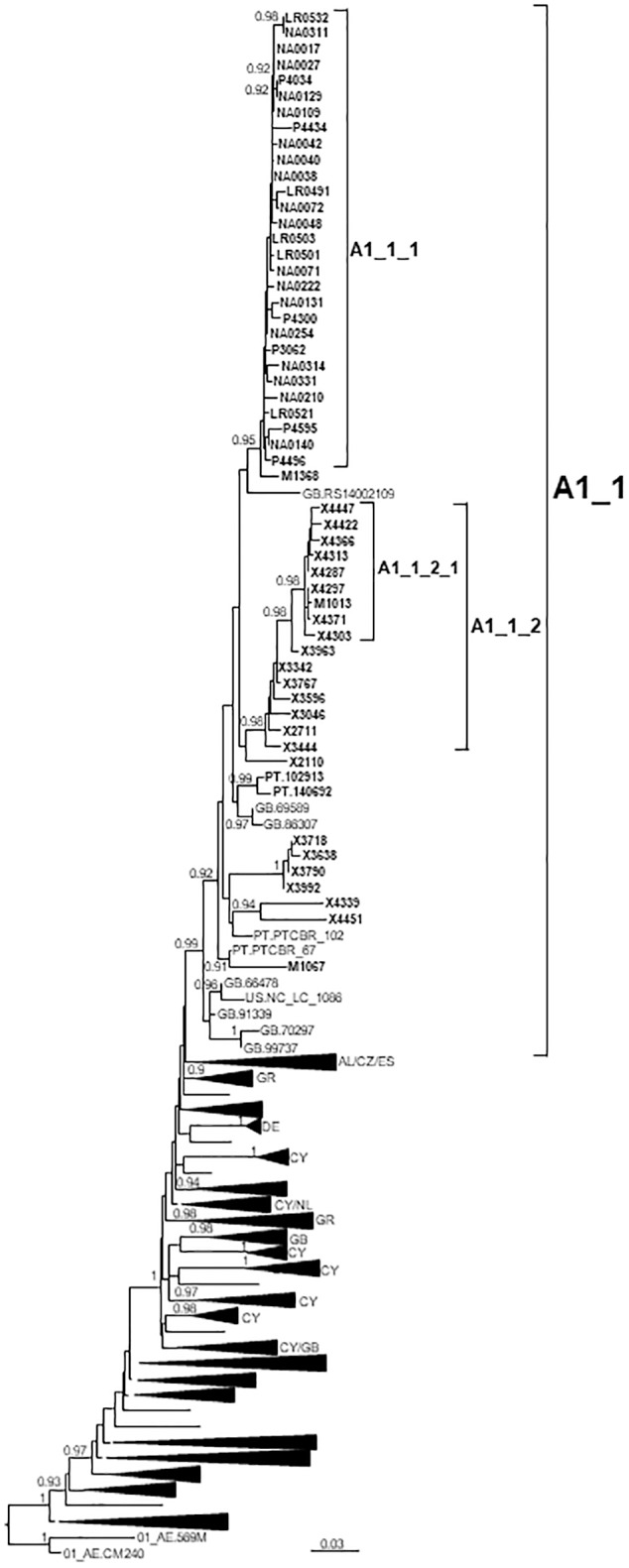
Maximum likelihood tree of PR-RT sequences of the A1_1 cluster. The tree was constructed with PhyML, with assessment of node support with the aLRT SH-like procedure. The analysis incorporates 338 A1 subsubtype PR-RT sequences from databases that in preliminary analyses with FastTree branched closer to the A1_1 cluster and from NFLG sequences, and two CRF01_AE sequences used to root the tree. For better viewing, clades outside of the A1_1 cluster are collapsed. Only aLRT SH-like node support values ≥ 0.90 are shown. Sequences obtained by us are in bold type. Sequences from databases are labeled with the two-letter ISO code of the country of sample collection followed by the sample name. Collapsed clades most closely related to the A1_1 cluster are labeled with the two-letter codes of the countries of sample collection, excluding countries represented by a single sequence.

A1_1 comprised 66 individuals, 54 studied by us and 12 with sequences deposited in databases (7 from United Kingdom, 4 from Portugal, and 1 from United States) (Figure 2). A1_1 was also related to viruses from the A1 subtype lineage circulating in Greece, Albania, and Cyprus (Ciccozzi et al., 2005; Paraskevis et al., 2007; Pineda-Peña et al., 2018). Samples from Spain were collected in 5 regions, mainly in Galicia and Navarre.

Forty five of 47 individuals with available data were diagnosed in 2012–2018.

Within A1_1, there were two main subclusters: A1_1_1, comprising all individuals from Navarre, Basque Country, and La Rioja, and 1 from Madrid; and, A1_1_2 comprising individuals mostly from Galicia, with a majority grouping in a subsubcluster.

Most (93%) individuals in the cluster were men, with predominance of MSM.

### F1_3 Cluster

**FIGURE 3:**
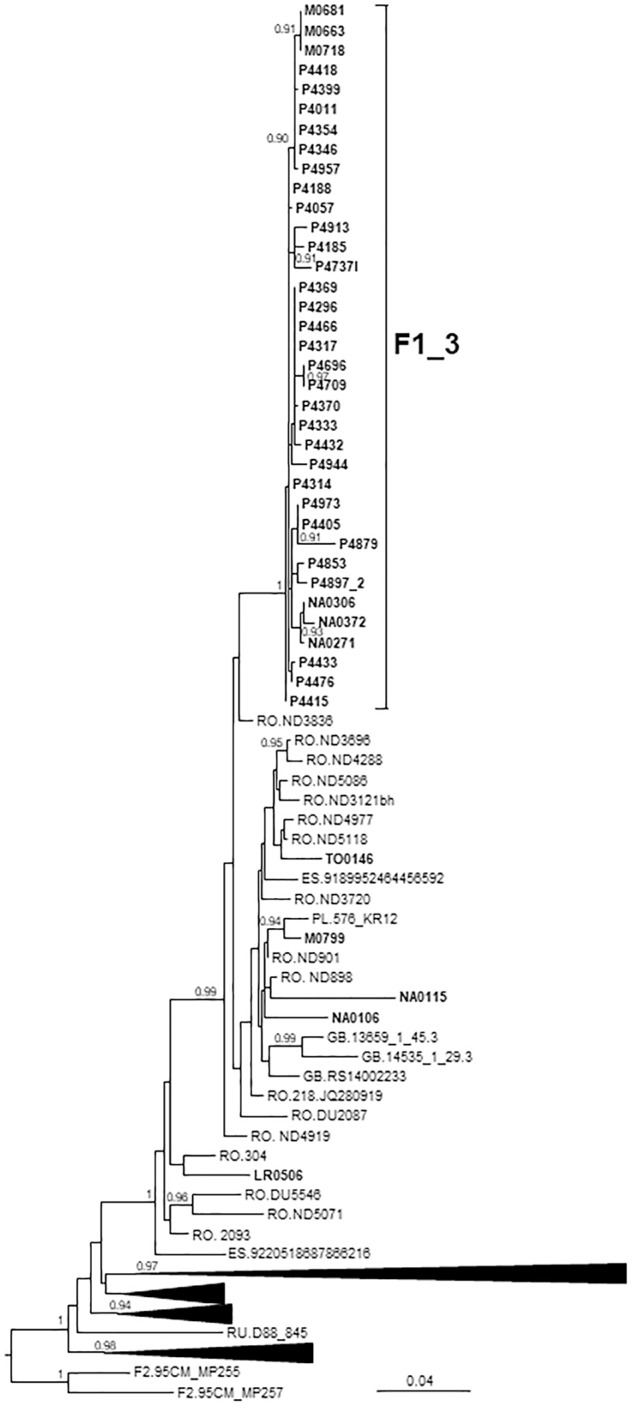
Maximum likelihood tree of PR-RT of viruses of the F1_3 cluster. The tree was constructed with PhyML, with assessment of node support with the aLRT SH-like procedure. The analysis incorporates 358 PR-RT F1 subsubtype sequences from databases that in preliminary analyses with FastTree branched closer to the F1_3 cluster and from NFLG sequences, and two F2 subsubtype sequences used to root the tree. For better viewing, clades outside of the F1_3 cluster, excluding those most closely related to the F1_3 cluster, are collapsed. Only aLRT SH-like node support values ≥ 0.90 are shown. Sequences obtained by us are in bold type. Sequences from databases are labeled with the two-letter ISO code of the country of sample collection followed by the sample name.

F1_3 comprised 36 individuals. There were no sequences from databases belonging to F1_3, but viruses from Romania, most of them transmitted sexually (Niculescu et al., 2015), were closely related to it (Figure 3). Five viruses from Spain (4 of them sequenced by us), 3 from United Kingdom, and 1 from Poland also branched close to F1_3, interspersed among the Romanian samples. Most F1_3 samples were from Basque Country.

All infections were diagnosed in 2014 or later. All individuals in F1_3 were men. Transmission route was sexual in all for which data were available, with a majority of MSM.

### C_7 Cluster

**FIGURE 4:**
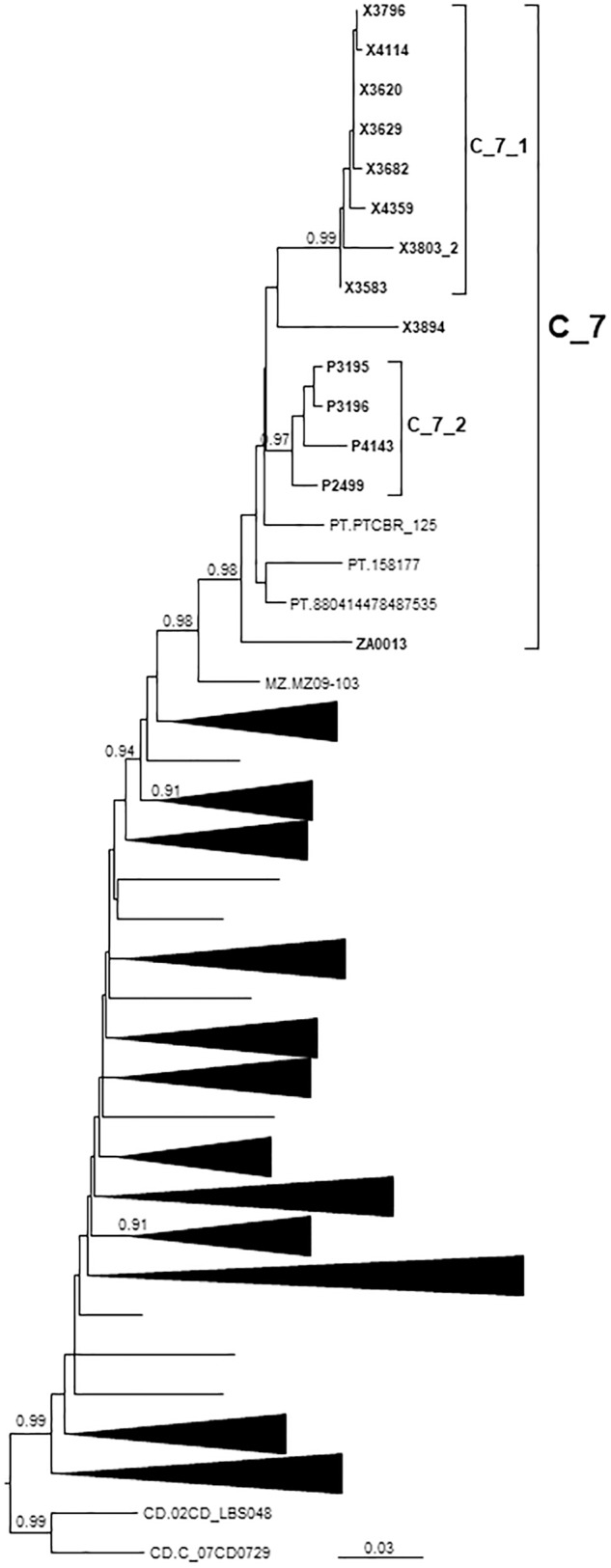
Maximum likelihood tree of PR-RT of viruses of the C_7 cluster. The tree was constructed with PhyML, with assessment of node support with the aLRT SH-like procedure. The analysis incorporates 312 PR-RT subtype C sequences from databases that in preliminary analyses with FastTree branched closer to the C_7 cluster and from NFLG sequences, and two subtype C sequences from the Democratic Republic of Congo used to root the tree. For better viewing, clades outside of the C_7 cluster are collapsed. Only aLRT SH-like node support values ≥ 0.90 are shown. Sequences obtained by us are in bold type. Sequences from databases are labeled with the two-letter ISO code of the country of sample collection followed by sample name.

C_7 comprised 17 individuals, including 14 studied by us and 3 from Portugal, whose sequences were retrieved from databases. One sequence from Mozambique branched basally to C_7 (Figure 4). C_7 comprised two subclusters, C_7_1, comprising all but one samples from Galicia, and C_7_2, comprising all 4 samples from Basque Country. All but one had been diagnosed in 2012–2017.

All but one Spanish samples were from Galicia or Basque Country. All individuals in C_7 were sexually infected men, with 5 being self-declared MSM.

### Bayesian Analyses and Temporal and Geographic Estimations

To estimate the temporal and geographic origins of clusters and subclusters, Bayesian coalescent analyses were performed with PR-RT sequences, summarizing the posterior distribution of trees with MCC trees. Prior to these analyses, temporal signal was analyzed, revealing a clock-like structure in all four datasets used for subsequent analyses (*r*^2^= 0.4395 in CRF02_1, *r*^2^= 0.5399 in A1_1, *r*^2^= 0.7175 in F1_3, and *r*^2^= 0.3518 in C_7), indicating that the datasets contained sufficient temporal structure for reliable estimation of divergence times.

In the Bayesian analyses, all four clusters previously defined via ML were supported by node PP values > 0.98.

**FIGURE 5:**
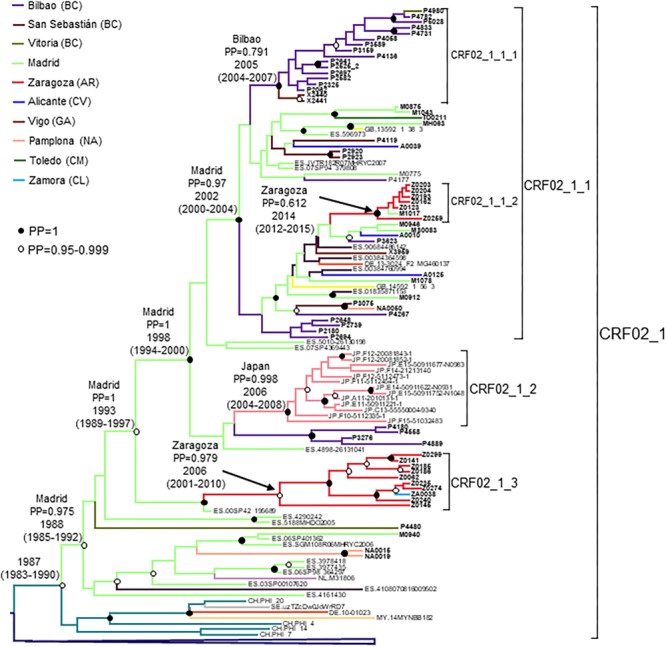
Maximum clade credibility tree of PR-RT sequences of the CRF02_1 cluster. The tree also includes 40 other PR-RT CRF02_AG sequences from databases. For better viewing, the clade comprising viruses branching outside of the CRF02_1 cluster is collapsed. Sequences obtained by us are in bold type. Sequences from databases are labeled with the two-letter ISO code of the country of sample collection followed by the sample name. Nodes supported by PP = 1 and PP = 0.95–0.999 are marked with filled and unfilled circles, respectively. Colors of terminal and internal branches represent sampling locations and most probable locations of the corresponding nodes, respectively, according to the legend on the left. For the nodes corresponding to CRF02_1 cluster and its major subclusters, the location with the highest PP (if > 0.5) and the tMRCAs (with 95% HPD intervals) are indicated above or close to the subtending branches.

The tMRCA of the entire CRF02_1 cluster was estimated around 1986, but the location of the MRCA was uncertain, since the most probable one was supported by a PP < 0.5. However, the location of the MRCA of the subcluster comprising all but the 8 most basal sequences (collected in Switzerland, Sweden, Germany, Malaysia, and Hong Kong), with tMRCA around 1988, had a strong support in Madrid, with a PP of 0.975 (Figure 5). Subcluster CRF02_1_1 emerged around 2002 in Madrid, CRF02_1_2 around 2006 in Japan, and CRF02_1_3 around 2006 in Zaragoza.

**FIGURE 6:**
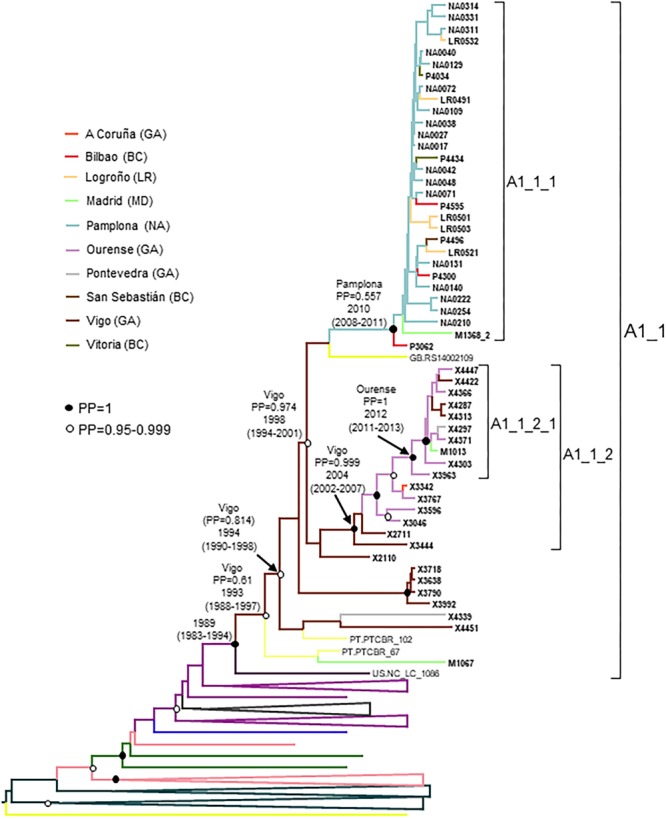
Maximum clade credibility tree of PR-RT sequences of the A1_1 cluster. The tree also includes a sequence from US that in ML trees branched close to the A1_1 cluster (samples from United Kingdom were excluded because no information on time of sample collection was available), and A1 subsubtype PR-RT sequences from NFLG sequences from databases. For better viewing, clades outside of the A1_1 cluster are collapsed. Sequences obtained by us are in bold type. Sequences from databases are labeled with the two-letter ISO code of country of sample collection followed by sample name. Clades most closely related to the A1_1 cluster are labeled with the two-letter ISO code of the sampling countries of viruses contained in it. Nodes supported by PP = 1 and PP = 0.95-0.999 are marked with filled and unfilled circles, respectively. Colors of terminal and internal branches represent sampling locations and most probable locations of the corresponding nodes, respectively, according to the legend on the left. For the nodes corresponding to the A1_1 cluster and its major subclusters, the location with the highest PP (if > 0.5) and the tMRCAs (with 95% HPD intervals) are indicated above or close to the subtending branches.

The estimated tMRCA of the entire A1_1 cluster was around 1989, but the location of the MRCA was uncertain, since the most probable one was supported by a PP < 0.5. However, the location of the MRCA of the subcluster comprising all but the 6 most basal sequences, with tMRCA around 1994, was supported by a PP of 0.814 in the city of Vigo, Galicia. The emergence of subcluster A1_1_1 was around 2010, with highest location PP in Pamplona, and that of A1_1_2 was around 2004 in Vigo, Galicia (Figure 6). It should be pointed out that 2 samples from Portugal and 6 from United Kingdom could not be used in the Bayesian analyses, since no collection year was available for them.

**FIGURE 7:**
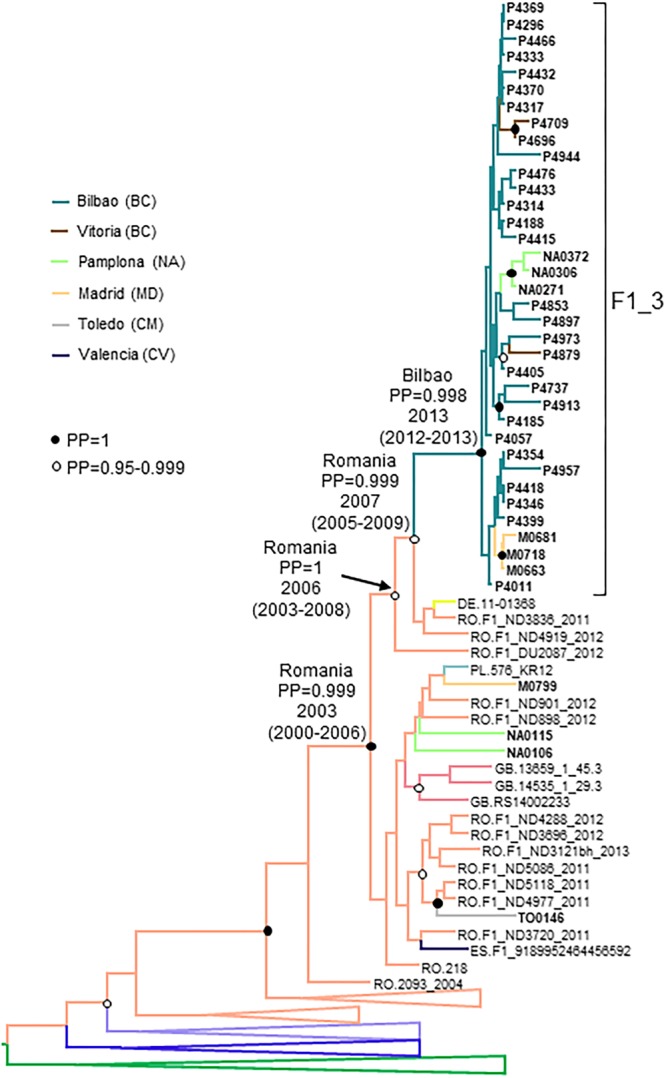
Maximum clade credibility tree of PR-RT sequences of the F1_3 cluster. The tree also includes F1 subsubtype sequences from databases branching close to the F1_3 cluster and F1_3 subsubtype PR-RT sequences from NFLG sequences from databases. For better viewing, clades outside of the F1 cluster are collapsed, except those most closely related to the F1_3 cluster. Sequences obtained by us are in bold type. Sequences from databases are labeled with the two-letter ISO code of country of sample collection followed by sample name. Nodes supported by PP = 1 and PP = 0.95–0.999 are marked with filled and unfilled circles, respectively. Colors of terminal and internal branches represent sampling locations and most probable locations of the corresponding nodes, respectively, according to the legend on the left. For the nodes corresponding to the F1_3 cluster and the clades within which it is contained, the location posterior probabilities and the tMRCAs (with 95% HPD intervals) are indicated above or close to the subtending branches.

The estimated tMRCA of F1_3 was around 2013 in the city of Bilbao, with a strongly supported ancestry in Romania (Figure 7).

**FIGURE 8:**
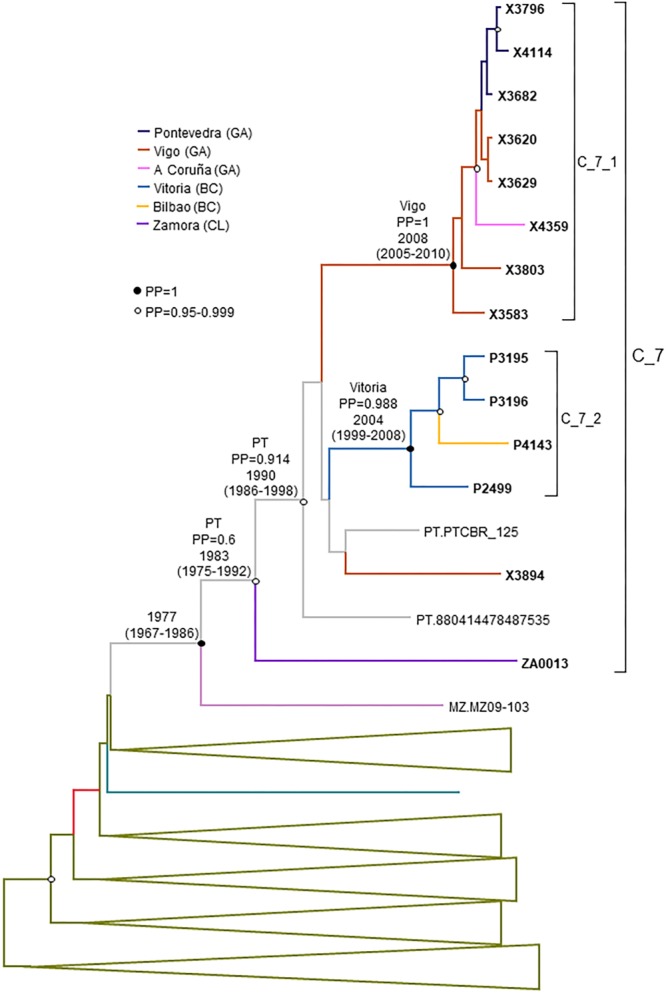
Maximum clade credibility tree of PR-RT sequences of the C_7 cluster. The tree also includes a subtype C sequences from Mozambique that in ML trees branched close to the C_7 cluster and subtype C PR-RT sequences from NFLG sequences from databases. For better viewing, clades outside of the C_7 cluster are collapsed. Sequences obtained by us are in bold type. Sequences from databases are labeled with the two-letter ISO code of country of sample collection followed by sample name. Nodes supported by PP = 1 and PP = 0.95–0.999 are marked with filled and unfilled circles, respectively. Colors of terminal and internal branches represent sampling locations and most probable locations of the corresponding nodes, respectively, according to the legend on the left. For the nodes corresponding to the C_7 cluster and subclusters within it, the location posterior probability and the tMRCAs (with 95% HPD intervals) are indicated above or close to the subtending branches. tMRCA is also indicated for the node corresponding to the clade including the sample from Mozambique (most probable location is omitted, since its PP is below 0.5).

Finally, the estimated tMRCA of C_7 was around 1983. Its most probable origin was Portugal, but with a PP support for the entire cluster of only 0.6. However, the location of the MRCA of the subcluster comprising all but the most basal sample had a strong support in Portugal (PP = 0.91) (Figure 8). Subcluster C_7_1 emerged around 2008 in Vigo and C_7_2 around 2004 in Vitoria, Basque Country.

### ARV Drug Resistance Mutations

In CRF02_1, 5 sequences (4 from Spain and one from Germany) had ARV drug resistance mutations. One was from a drug-experienced individual in therapeutic failure with multiple drug resistance mutations, and two, with K101E and K103N, respectively, mutations of resistance to non-nucleoside reverse transcriptase inhibitors (NNRTI), were from drug-naïve individuals. The other two, with K103N and K101E mutations, respectively, were from database sequences without data on drug treatment. In A1_1, one database sequence from United Kingdom had Y188C and G190A NNRTI resistance mutations. In C_7, all but 2 sequences had L90M mutation associated with protease inhibitor drug resistance; all 13 Spanish sequences with this mutation were from drug-naïve individuals.

### Near Full-Length Genome Sequences

**FIGURE 9:**
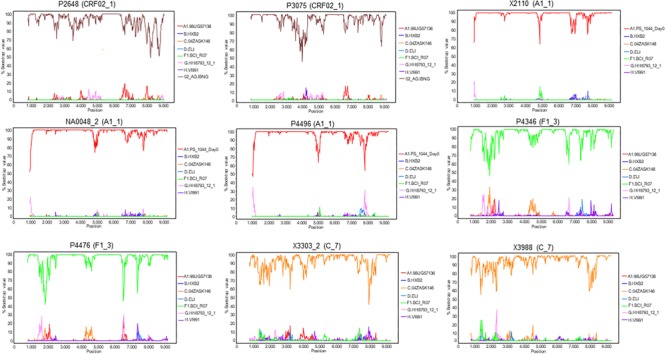
Bootscan analyses of NFLG sequences of viruses from the identified clusters. Virus names, with cluster in parentheses, are above each plot. A window of 400 nt was used for viruses of the A1_1, F1_3, and C_7 clusters, and of 600 nt for viruses of the CRF02_1 cluster, sliding in 20 nt increments. The horizontal axis represents the nucleotide position in the HXB2 proviral genome of the window’s midpoint. The vertical axis represents the bootstrap value supporting clustering with reference sequences, listed on the right of each plot.

To determine whether the viruses from the clusters were of uniform genetic form all along their genomes or were interclade recombinants, two NFLG sequences were obtained for each cluster, either from plasma RNA (P2648, P3075, P4496, P4346, and P4476) or from RNA extracted from culture supernatant (NA0048_2, X3303_2, and X3988). An additional NFLG from the A1_1 cluster (X2110, GenBank accession FJ670523) had been obtained previously by us (Cuevas et al., 2010). Bootscan analyses showed that all were of uniform genetic form along their genomes (Figure 9). We note that NFLG sequences of two CRF02_1 viruses from United Kingdom (Figure 1) are also available at sequence databases (Yebra et al., 2018; HIV Sequence Database, 2019) (GenBank accessions MF109381, MF109550).

## Discussion

In Western Europe, subtype B has been largely predominant among MSM since the early HIV-1 epidemic, but in recent years other genetic forms have been reported to be circulating in this population, as evidenced by their grouping in phylogenetic clusters comprising mostly European individuals. In this study, based on a large dataset from Spain, we found an increase in proportions of non-subtype B infections among MSM in recent years (Supplementary Figure 2) and higher clustering frequency in MSM compared to heterosexually infected individuals (Supplementary Figure 1). Among clusters associated with MSM, five large ones were of non-subtype B genetic forms, one of which, of F1 subtype, was reported previously (Thomson et al., 2012; Delgado et al., 2015). The other four were of CRF02_AG and subtypes A1, F1, and C, for which here we analyze epidemiological correlations, estimated emergence times and places, NFLGs, and drug resistance mutations.

The CRF02_AG cluster (CRF02_1) comprised 115 individuals, including 67 studied by us and 48 whose sequences were retrieved from databases, making it one of the largest non-subtype B clusters circulating among MSM reported to date in Western Europe (Delgado et al., 2015; Vinken et al., 2017). CRF02_AG is the predominant HIV-1 genetic form in most West African countries (Montavon et al., 2000; Hemelaar et al., 2018) and is common in West-Central Africa. It also propagates as a minor form in several Western European countries (Giuliani et al., 2013; Brand et al., 2014; Tamalet et al., 2015; Beloukas et al., 2016; Chaillon et al., 2017; Verhofstede et al., 2018), Tunisia (El Moussi et al., 2017), and Brazil (Delatorre et al., 2012), and in 2002 caused an outbreak among PWID in Uzbekistan (Carr et al., 2005), with subsequent dissemination to Kazakhstan (Eyzaguirre et al., 2007; Lapovok et al., 2014) and Russia (Moskaleychik et al., 2015), giving rise to CRF63_02A1 through recombination with the former Soviet Union subtype A variant (Baryshev et al., 2014; Shcherbakova et al., 2014). CRF02_AG has been reported to be one of the most common non-subtype B genetic forms in Western Europe (together with subtypes A1 and C) (Abecasis et al., 2013; Beloukas et al., 2016; Hemelaar et al., 2018) and in Spain (Yebra et al., 2012). The CRF02_AG cluster here described is not completely new, since a cluster of four individuals from the region of Valencia belonging to it was reported by other authors, who also noted that 9 sequences from databases, 7 from Spain and 2 from Ecuador, were related to the Valencian cluster (Bracho et al., 2014). However, the data here presented considerably enlarge the size and the geographic range of the cluster. CRF02_1 comprises viruses from 8 Spanish regions and from 9 other countries, from Western Europe, Asia and South America, with 13 Japanese viruses grouping in a monophyletic subcluster, indicating that it is circulating in this country. Although CRF02_1 is mainly associated with MSM, a subcluster comprising 11 individuals, 10 of them from the city of Zaragoza, propagates via heterosexual contact (Table 1). The origin of CRF02_1 is not recent, with a tMRCA estimated around 1986, with uncertain location, for the entire cluster, or 1988 in Madrid for the subcluster excluding the 8 most basal sequences, but its three major subclusters emerged in recent years, with tMRCAs in the 2000s.

The A1 subtype cluster (A1_1) is the second largest cluster here described, with 66 individuals, 54 studied by us and 12 with sequences in databases. A1 subtype circulates mainly in Eastern, Central and Western Africa (Hemelaar et al., 2018), all former Soviet Union (FSU) countries (Bobkova, 2013), Greece (Paraskevis et al., 2007), Albania (Ciccozzi et al., 2005), and Cyprus (Pineda-Peña et al., 2018), and as a minor form in India (Pandey et al., 2016), although some authors designate the variants circulating in Western Africa and FSU as distinct subsubtypes (A3 and A6, respectively) (Meloni et al., 2004; Foley et al., 2016). The lineage circulating in Greece and Albania, also detected in Cyprus, is of monophyletic origin, with estimated tMRCA around 1978 (Paraskevis et al., 2007). A1 subtype clusters have been reported in United Kingdom (Gifford et al., 2007; Hughes et al., 2009; Ragonnet-Cronin et al., 2016), Italy (Lai et al., 2016), Switzerland (von Wyl et al., 2011), and Portugal (Carvalho et al., 2015), but the one here reported is the largest reported to date in Western Europe. A1_1 comprises individuals from 5 Spanish regions and 3 other countries (United Kingdom, Portugal, and United States), and is related to the Greek-Albanian A1 lineage (Figure 2). Its origin is not recent, with estimated tMRCA around 1989, with uncertain location for the entire cluster, or around 1994 in Vigo, Galicia, excluding the 6 most basal sequences, but its two major subclusters are of recent origin, with tMRCAs around 2004 and 2010, respectively.

The F1 cluster (F1_3) comprises 36 individuals, all resident in Spain, most of them in Bilbao, Basque Country. Of the clusters here described, this is the one with the most recent origin, with estimated tMRCA around 2013 in Bilbao. It is currently increasing in size, with 6 individuals newly diagnosed in 2018. Subtype F1 is circulating in Central Africa, Brazil and Romania (Dumitrescu et al., 1994; Louwagie et al., 1994; Bandea et al., 1995; Apetrei et al., 1997; Op de Coul et al., 2000; Hemelaar et al., 2018), and F1 subtype clusters have been recently identified in Spain (Thomson et al., 2012; Delgado et al., 2015), Belgium (Vinken et al., 2017; Verhofstede et al., 2018), Switzerland (Castro et al., 2010), Italy (Lai et al., 2012), and Portugal (Carvalho et al., 2015). F1_3 is most closely related to F1 viruses from Romania (Figure 4), which are related to viruses circulating in Angola (Guimarães et al., 2009) and initially propagated among adults via sexual contact, with subsequent propagation among institutionalized children through contaminated injection equipment (Op de Coul et al., 2000; Bello et al., 2012), and more recently among PWID (Temereanca et al., 2013; Niculescu et al., 2015). The expansion of an F1 subtype cluster of Romanian ancestry in Spain has its counterpart in the recent expansion of CRF14_BG, originally described in Spain (Thomson et al., 2001; Delgado et al., 2002) and Portugal (Esteves et al., 2003; Abecasis et al., 2011; Bartolo et al., 2011), among PWID in Romania (Niculescu et al., 2015). The exchange of HIV-1 genetic forms between Romania and Spain reflects the presence of a large Romanian immigrant population in Spain (Instituto Nacional de Estadística, 2018), frequently traveling between both countries.

The subtype C cluster (C_7) comprises 17 viruses, 14 from three Spanish regions, predominantly from Galicia, and three from Portugal. It comprises a Galician and a Basque subclusters (Figure 4). This is not the first report of this cluster, as it was described by us when it comprised only 7 individuals, within the context of the description of HIV-1 clusters bearing ARV drug resistance mutations, noting that viruses belonging to it carry the L90M mutation of resistance to protease inhibitors (Vega et al., 2015). This mutation was found in 15 of 17 C_7 viruses. Subtype C is the most prevalent clade in the HIV-1 pandemic, circulating mainly in Southern and Eastern Africa, Southern Brazil, and South Asia (Hemelaar et al., 2018). In Western Europe, subtype C clusters have been reported in the United Kingdom (Hughes et al., 2009; de Oliveira et al., 2010; Ragonnet-Cronin et al., 2016), Italy (Lai et al., 2014), and Portugal (Carvalho et al., 2015).

The origin of C_7 is not recent, with a tMRCA around 1983, with a most probable origin in Portugal, but the tMRCAs of its subclusters are relatively recent, in 2008 and 2004, respectively. C_7 is related to a virus from Mozambique, a former Portuguese colony where subtype C is the predominant HIV-1 genetic form (Bellocchi et al., 2005).

It should be noted that 3 individuals in each of clusters F1_3 and C_7, composed entirely of men, declared being heterosexual, and that in clusters CRF02_1 (excluding the heterosexual-associated CRF02_1_3 subcluster) and A1_1, among sexually infected individuals the number of self-declared heterosexual men exceeds the number of women (8 vs. 3 in CRF02_1 and 6 vs. 3 in A1_1). This suggests that at least some of the self-declared heterosexual men within the clusters could in fact be MSM, who, due to social stigma and discrimination, do not declare their real sexual behaviors, as suggested by other authors who found similar discrepancies between self-reported heterosexual behavior and phylogenetic clustering with sequences from MSM (Hué et al., 2014; Hoenigl et al., 2016; Ragonnet-Cronin et al., 2018).

The descriptions of the four clusters here analyzed is in line with those of other non-subtype B clusters reported to have expanded among MSM in Western Europe. However there are some salient features of the clusters here described that should be highlighted: first, the relatively large size of CRF02_1_1 and A1_1, greater than most non-subtype B clusters reported among MSM in Western Europe; second, their wide geographic distribution among different countries, which contrasts with the predominantly within-country clustering found by other authors in Europe (Frentz et al., 2013; Paraskevis et al., 2019); and, third, the rapid expansion of F1_3, with 36 diagnoses in only 4 years.

The expansion of large clusters among MSM in recent years, as here reported, reflects the existence of high risk sexual behaviors, which should prompt public health authorities to implement public health measures aimed at preventing HIV-1 transmission in this population, including behavioral interventions to reduce risky practices, preexposure prophylaxis (Grant et al., 2010; Volk et al., 2015; McCormack et al., 2016), and early diagnosis and treatment of HIV-1 infections (European Centre for Disease Prevention and Control, 2015; United Nations Population Fund, the Global Forum on MSM and HIV, United Nations Development Programme, World Health Organization, United States Agency for International Development, and World Bank, 2015). Prevention of HIV-1 transmission among MSM could also result in a reduction of heterosexually-transmitted infections, which can have their source in MSM networks, as seen in subcluster CRF02_1_3, associated with heterosexual transmission, and as reported by other authors (Oster et al., 2015; Esbjörnsson et al., 2016).

Continued HIV-1 molecular surveillance will be necessary to gain insight in real time on the dynamics of expansion of transmission networks, which will allow to focus prophylactic efforts in populations with the highest risk of HIV-1 acquisition and ongoing transmission (Little et al., 2014; Wang et al., 2015; Poon et al., 2016; Ratmann et al., 2016; Brenner et al., 2017; Chaillon et al., 2017; German et al., 2017; Oster et al., 2018; Wertheim et al., 2018) and to monitor the efficacy of public health interventions aimed at controlling the epidemic (Wertheim et al., 2011; Magiorkinis et al., 2018). HIV-1 molecular surveillance can also provide important information for the design of vaccine immunogens adapted to the major HIV-1 variants actively propagating in different areas, considering the correlation of HIV-1 clades to susceptibility to protective immune responses (Cao et al., 2000; Thomson et al., 2002; Binley et al., 2004; Geldmacher et al., 2007; Seaman et al., 2010; Hraber et al., 2014), and with potential to induce broadly neutralizing antibody responses (Kouyos et al., 2018), and will allow to obtain reagents derived from these variants for use in vaccine-related research (Cuevas et al., 2010; Revilla et al., 2011; Hora et al., 2016). These reagents will also be useful for studies on the biological basis of increased pathogenicity (Baeten et al., 2007; Kiwanuka et al., 2010; Li et al., 2014; Pérez-Álvarez et al., 2014; Kouri et al., 2015; Venner et al., 2016) and transmissibility (Kiwanuka et al., 2009) and diminished response to ARV drugs (Pernas et al., 2014; Cid-Silva et al., 2018) exhibited by some HIV-1 variants.

## Data Availability

The datasets generated for this study can be found in GenBank, MK177651–MK177824, MK177825–MK177829, KT276258, KY496622, and KY989952.

## Ethics Statement

This study was carried out in accordance with the recommendations of ’name of guidelines, name of committee’ with written informed consent from all subjects. All subjects gave written informed consent in accordance with the Declaration of Helsinki. The protocol was approved by the Bioethics and Animal Well-being Committee of Instituto de Salud Carlos III, Majadahonda, Madrid, Spain.

## Author Contributions

MT, ED, and LP-Á conceived the study and supervised the experimental work. ED, MT, MC, AF-G, FD-F, JC, JM-L, and MS processed sequences and performed phylogenetic analyses. MT and FD-F performed phylodynamic analyses. HG performed data curation and phylogenetic analyses. SB, VM, AF-G, MS-M, EG-B, and CC performed experimental work. The members of the Spanish Group for the Study of New HIV Diagnoses recruited patients and obtained epidemiological data. MT wrote the manuscript with contributions from the other authors.

## Conflict of Interest Statement

The authors declare that the research was conducted in the absence of any commercial or financial relationships that could be construed as a potential conflict of interest.

## Footnotes

1 http://tree.bio.ed.ac.uk/software/figtree/

2 http://tree.bio.ed.ac.uk/software/tracer/

## Abbreviations

MCC: maximum clade credibility
MCMC: Markov chain Monte Carlo
ML: maximum likelihood
MRCA: most recent common ancestor
MSM: men who have sex with men
NFLG: near full-length genome
PP: posterior probability
PR-RT: protease-reverse transcriptase
PWID: people who inject drugs
tMRCA: time of most recent common ancestor
