# Supplementary figures and images for: Diverse Large HIV-1 Non-subtype B Clusters Are Spreading Among Men Who Have Sex With Men in Spain
Source: Front Microbiol. 2019 Apr 3;10:655. doi: 10.3389/fmicb.2019.00655 (PMC6457325; doi:10.3389/fmicb.2019.00655)

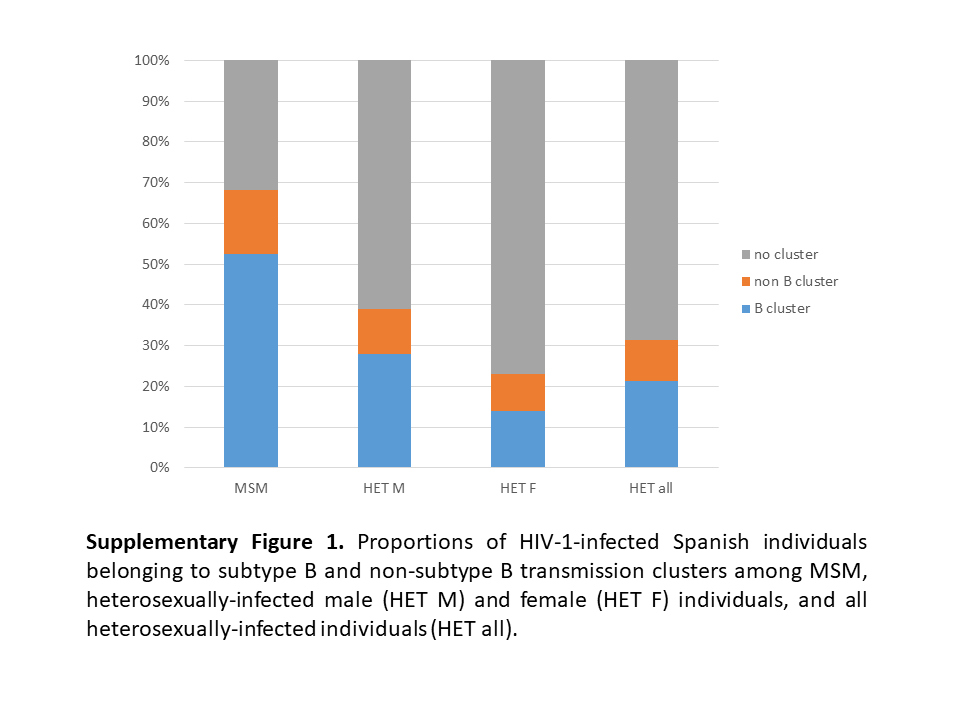

Supplement: Supplementary file 2 [file Image_1.TIFF]

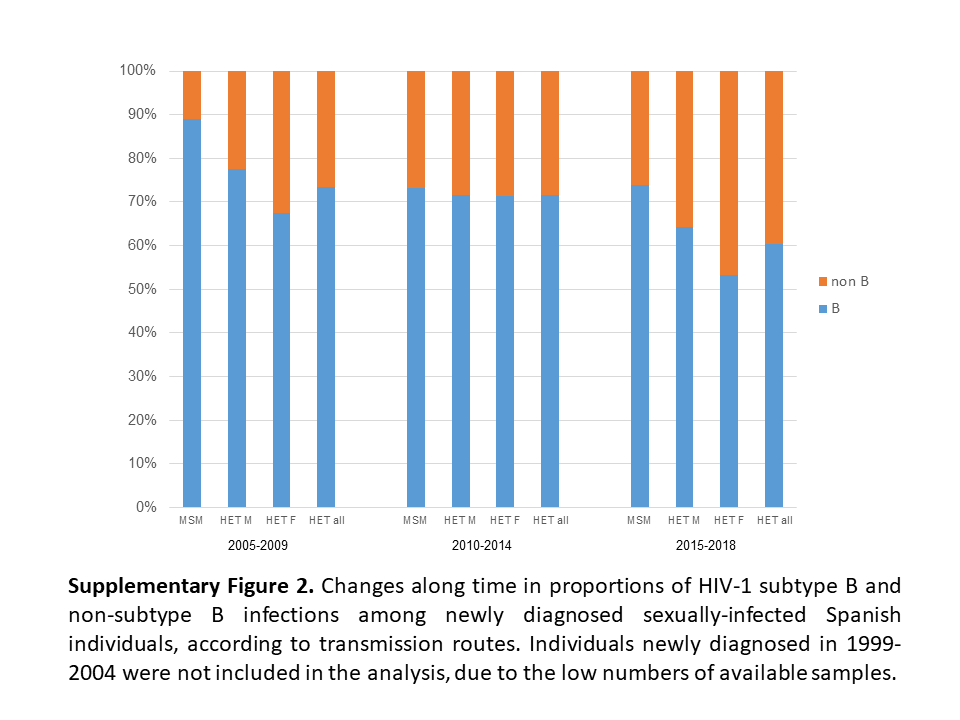

Supplement: Supplementary file 3 [file Image_2.TIFF]
